# Coded Aperture Optimization in X-Ray Computed Tomography via Sparse Covariance Matrix Estimation

**DOI:** 10.3390/s25247479

**Published:** 2025-12-09

**Authors:** Yuqi Jiang, Tianyi Mao, Jianyong Zhou, Qile Zhao, Jun Yin, Xuedong Yi, Haiyou Wu

**Affiliations:** 1 The 44th Research Institute of China Electronics Technology Corporation, Chongqing 400060, China; 2 The 24th Research Institute of China Electronics Technology Corporation, Chongqing 400060, China; 3 College of Automation, Nanjing University of Posts and Telecommunications, Nanjing 210023, China; 4 Technology Department of Unmanned Platform System, Xi’an Institute of Applied Optics, Xi’an 710065, China; 5 Department of Automation, Tsinghua University, Beijing 100081, China

**Keywords:** computed tomography, coded aperture, optimization, covariance matrix

## Abstract

Coded aperture X-ray computed tomography (CAXCT) measures coded X-ray projections to reconstruct the inner structure of an object. Coded apertures, which determine the point spread function, can be designed to improve the reconstruction quality, but most approaches are computationally expensive, leading to very small images. In this paper, a sparse covariance matrix estimation approach is introduced to minimize the information loss sensed by projections corresponding to large tomographic images. The covariance matrix representing the map of the overlapping information of the projections is obtained by using block matrix multiplication and sparse estimation. A heuristic variant algorithm with a noise factor is presented to search the combinations of *D* projections leading to maximum non-overlapping information acquisition, where *D* is the number of unblocking elements on the coded apertures. Numerical experiments with simulated datasets show that the optimization performance of the proposed method is comparable to that of state-of-the-art methods with small images. Further, for the analyzed cases, coded aperture optimization was performed with 512 × 512 images by analyzing coefficients smaller than 0.02% in the covariance matrix.

## 1. Introduction

X-ray computed tomography (CT) is a non-invasive imaging technique to reconstruct high-resolution three-dimensional (3D) images from tomographic measurements [1]. It has been extensively used in medical/biological imaging, security inspection, non-destructive testing and so on [2,3]. An iterative algorithm, the algebraic reconstruction technique (ART), was earlier used to reconstruct tomographic images from parallel-beam and fan-beam projections [4]. It was soon replaced by filtered back-projection (FBP) algorithms based on an analytical formulation due to their computational efficiency and low memory demands [4]. However, numerous X-ray projections with redundant measurements are required in FBP algorithms, leading to a high radiation dose, possibly damaging the specimen [5]. With the development of computational ability and signal processing in recent years, iterative reconstruction algorithms, for example, sparsity-exploiting based compressed sensing (CS), have been proposed to reduce the number of projections in the cases of limited and sparse view angles [6]. However, the redundant projections from a particular X-ray source are not considered in the uniform sampling of sparsity-exploiting methods and thus fall short in obtaining high quality reconstructions as the number of projections is further reduced. Coded aperture X-ray computed tomography (CAXCT) has been introduced to non-uniformly sample the object with binary coded apertures placed in front of the X-ray sources, reducing the number of X-ray projections while simultaneously improving the reconstruction quality [7].

The coded aperture patterns, besides the projection geometry and number of view angles, are the only varying component in CAXCT to affect the point spread function (PSF) of the system and in turn determine the attainable imaging quality and radiation dose level. However, the structure matrix in CAXCT is highly structured, and random coded aperture cases are not optimal in general CS scenarios. A uniform sensing strategy was initially proposed to optimize the coded apertures, where the elements on the detector and object are designed to be sensed uniformly [8,9,10]. The direct binary search (DBS) algorithm is used to obtain the uniform complementary coding, but the method is empirical, with no theoretical proof of the criterion used. An alternative gradient descent approach based on the restricted isometry property (RIP) condition and mutual coherence is presented for fan-beam projection systems in [11] and cone-beam projection systems in [12], respectively. The Gram matrix in the method is computationally expensive, and thus the radii of the Gershgorin Theorem are used to estimate the eigenvalue bounds at lower computational requirements without actually calculating the eigenvalues [13]. An efficient coded aperture optimization approach has been introduced to obtain the maximum non-overlapping information acquisition, leading to orders of magnitude faster computation [14,15]. The metric used in the gradient approach, the Gram matrix, is replaced by the covariance matrix, and thus the computational complexity is significantly reduced. However, the Gram matrix and covariance matrix representing the mutual coherence and the amount of information are usually dense matrices, and thus these approaches with significant gains are limited to 128 × 128 tomographic images due to the memory limitation.

This paper introduces a variant approach based on minimizing the overlapping information for high-resolution images. The covariance matrix, which represents the overlapping information of the projections, is sparsely estimated with distributed computing implementation. Then, the combinations of projections corresponding to unblocking elements can be obtained by analyzing the entire non-zero coefficients in the sparse covariance matrix. The results using simulated datasets show gains up to 3.3 dB by analyzing coefficients smaller than 0.02% in the covariance matrix corresponding to 512 × 512 images. This paper is organized as follows. Section 2 introduces the discrete forward model of the CAXCT system with fan-beam projection geometry. Section 3 presents the coded aperture design based on minimizing the overlapping information where the Pearson product moment correlation coefficient (PPMCC) matrix is used as the metric. The coefficients in the PPMCC matrix for large structure matrices are obtained by using block matrix multiplication and sparse estimation. In Section 4, the results using simulated datasets are presented for images with 128 × 128, 256 × 256 and 512 × 512 pixels. Finally, conclusions are given in Section 5.

## 2. Forward Model

**Figure 1:**
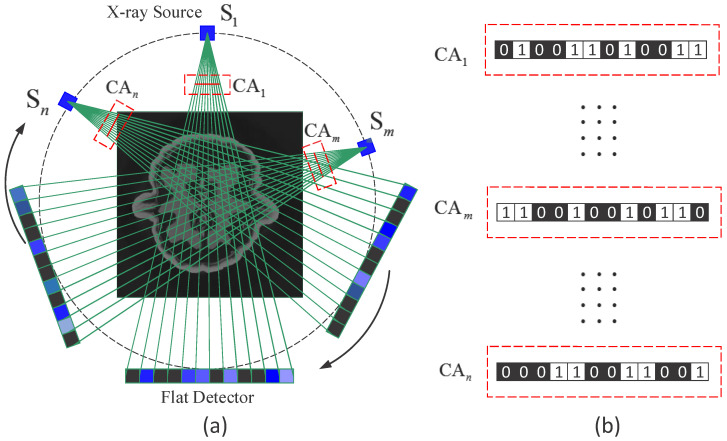
Optical setup of the CAXCT system. Reproduced from [14]. © 2018 Optical Society of America under the terms of the OSA Open Access Publishing Agreement. (**a**) The object is illuminated by coded fan-beam X-ray sources at *P* positions $\left[S_{1},S_{2},\ldots ,S_{P}\right]$, and the coded projections are captured by a flat detector. Part of the X-ray radiation is blocked by the blocking elements on the coded apertures, and the correspondent pixels on the flat detector are discarded. (**b**) CA represents the coded aperture, and the white and black squares represent unblocking and blocking elements, respectively.

As depicted in Figure 1, the object is illuminated by X-ray sources at a series of view angles, and the corresponding coded apertures are placed in front of the sources. Most of the X-ray projections are blocked by the blocking elements on the coded apertures, and thus the radiation dose is significantly reduced. The coded projections at each view angle are measured by a flat linear detector. Note that the elements on the coded apertures have one-to-one correspondence to those on the detectors. The transmission model of a monochromatic X-ray path *l* to a detector is given by the Beer–Lambert law [16].

(1) $$I^{l}=I_{0}^{l}\exp\left\{-{ʃ}_{0}^{\infty }\alpha (l)dl\right\},$$

where $I^{l}$ is the measurement on the detector, $I_{0}^{l}$ is the intensity of the monochromatic X-ray source and α is the linear attenuation coefficient varying in the *l* path.

Suppose $\vec{s}$ and $\vec{\theta }$ represent the location of the X-ray source and the direction of the projection, respectively. The measurements of the detector can be re-written as

(2) $$I\left(\vec{s},\vec{\theta }\right)=I_{0}\exp\left\{-{ʃ}_{0}^{\infty }f\left(\vec{s}+l\vec{\theta }\right)dl\right\},$$

where *f* represents the linear attenuation coefficient map of the object.

Due to the pixelated nature of detectors, the *i*th measurements in the matrix notation are given by

(3) $$I_{i}=I_{0}\exp\left\{-W_{i}f\right\},$$

where $I_{i}\in R^{M}$ denotes the vectorized representation of the *i*th measurements, $f\in R^{N}$ denotes the vectorized representation of the linear attenuation coefficient map and $W\in R^{M\;\times \;N}$ denotes the structure matrix, which represents the X-ray path characteristics of the ray projections from the source at a particular view angle to the corresponding detector. *M* and *N* are the resolution of the detector and object, respectively.

The *i*th coded apertures $c_{i}\in R^{M}$, i=1,2,…,P are placed in front of the sources at *P* view angles, and the *i*th measurements can be formulated as

(4) $$\begin{array}{cc} I_{i} & =c_{i}⊗I_{0}\exp\left\{-W_{i}f\right\}\\ \; & =I_{0}\exp\left\{-c_{i}⊗W_{i}f\right\}\\ \; & =I_{0}\exp\left\{-\mathrm{diag}\left(c_{i}\right)W_{i}f\right\}, \end{array}$$

where ⊗ and diag(·) represent element-wise multiplication and a diagonal matrix, respectively. Thus, the logarithmic measurements at *P* view angles can be re-written as

(5) $$y=\ln\left(\frac{I_{0}}{I}\right)=\mathrm{CWf},$$

where the coded aperture matrix C is defined as

(6) $$C=\left[\begin{array}{cccc} \mathrm{diag}(c_{1}) & 0 & \ldots & 0\\ 0 & \mathrm{diag}(c_{2}) & \ldots & 0\\ \ldots & \ldots & \ldots & \ldots \\ 0 & 0 & \ldots & \mathrm{diag}(c_{P}) \end{array}\right],$$

$I=[I_{1}^{T},I_{2}^{T},\ldots ,I_{P}^{T}]$ denotes the vectorized representation of the measurements at *P* view angles, $y\in R^{MP}$ denotes the vectorized representation of the corresponding logarithmic measurements at *P* view angles and $W=[W_{1}^{T},W_{2}^{T},\ldots ,W_{P}^{T}]$ denotes the structure matrix at *P* view angles. Note that the coded aperture matrix $C\in R^{MP\;\times \;MP}$ is a diagonal matrix, and all entries in $0\in R^{M\;\times \;M}$ are zero.

As depicted in Equation (5), the coded aperture matrix C functions as a downsampling operator, and the rank of the measurement matrix CW is determined by the number of non-zero entries. In order to reduce the radiation dose as much as possible, the number of non-zero entries is less than that of columns, and thus the forward model is an ill-conditioned problem which can be reconstructed by using CS. In most cases, the natural images f are sufficiently sparse in some bases $\Psi \in R^{N\;\times \;N}$, and such bases are partially incoherent with the measurement matrix CW. Then the equation can be re-written as y=CWΨθ=Aθ, where $\theta \in R^{N}$ is the sparse representation of f, and $A=\mathrm{CW}\Psi \in R^{N\;\times \;N}$ is the sensing matrix. The sparse representation can be obtained by solving the nonlinear optimization problem,

(7) $$\hat{\theta }=\underset{\theta }{\arg\min}\;{∥y-A\theta ∥}_{2}^{2}+\lambda {∥\theta ∥}_{1},$$

where λ is the regularization constant, and ${∥\cdot ∥}_{1}$ and ${∥\cdot ∥}_{2}$ represent the ${𝓁}_{1}$ and ${𝓁}_{2}$ norms, respectively.

## 3. Coded Aperture Optimization

### 3.1. Sparse Correlation Estimation

The upper bound of the reconstruction quality with CS-based algorithms is determined by the property of sensing matrix, and an important metric used in the design of the sensing matrix is the RIP condition given by [17]

(8) $$\left(1-\delta_{q}\right){∥\theta ∥}_{2}^{2}\leq ∥A\theta ∥\leq \left(1+\delta_{q}\right){∥\theta ∥}_{2}^{2},$$

where $\delta_{q}\in \left[0,1\right)$ is a constant and *q* is the number of non-zero elements in the sparse representation θ. Since the structure matrix W is highly structured, it is natural that the sensing matrix with random coded apertures only partially satisfies the RIP condition, and the reconstruction quality is inferior to that using an ideal sensing matrix such as a Gaussian matrix. A gradient descent approach based on the RIP condition is presented to optimize the coded apertures and reduce the constant $\delta_{q}$. However, the computational complexity of using the Gram matrix to estimate the RIP condition limits the image size to up to 128 × 128. In our previous work, non-overlapping information acquisition was introduced as an alternative metric to significantly improve the efficiency in obtaining the optimized coded apertures. The entries of the system matrix are attenuation coefficients describing ray projections from source to detector, i.e., measurements collected along X-ray paths. From this perspective, variables map to observations, while ray paths serve as features. Therefore, the measurement matrix CW represents the dimensionality reduction of features, and the purpose of designing the coded apertures is to obtain the maximum non-overlapping information carried by *D* X-ray projections, where *D* is the number of unblocking elements on the coded apertures. The cost function based on non-overlapping information acquisition is given by [14]

(9) $$\arg\min\sum_{i\neq j}Q_{ij}^{2}\;s.t.\;C_{ii}=C_{jj}=1\;\forall i,j,$$

and

(10) $$Q_{ij}=\frac{E[\left(W\left(i\right)-\mu_{i}\right)\left(W\left(j\right)-\mu_{j}\right)]}{\sigma_{i}\sigma_{j}}$$

where E[·] denotes the expected value; μ and σ denote the mean and standard deviation of a particular vector, respectively; and W(i) and W(j) denote the columns of the transpose of the structure matrix $W^{T}$. Note that $Q_{ij}$ is the (i,j)th element in the PPMCC matrix.

**Figure 2:**
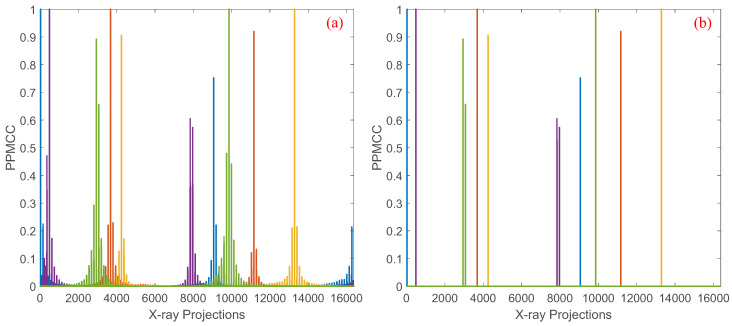
(**a**) shows the dense PPMCC matrix computed using all coefficients, and (**b**) shows the sparse PPMCC matrix obtained by applying a threshold of 0.5 for 64 × 64 images.

Limited by the memory of regular platforms, the previous algorithms solving Equations (9) and (10) perform well for *N* up to $128^{2}$, that is, the object with 128 × 128 pixels. For an object with 256 × 256 pixels, a separated method which divides the structure matrix into multiple sub-matrices is introduced to optimize the coded apertures, but only $1/L^{2}$ coefficients of the PPMCC matrix are considered in the optimization. The PPMCC matrix of the separated method with L=4, $N=64^{2}$, M=64 and P=64 is depicted in Figure 2b. Compared with the original PPMCC matrix shown in Figure 2a, only four squares along the diagonal vector are considered in the optimization, and thus the performance of optimization is inferior to that with smaller images.

However, it is difficult to directly use the maximum non-overlapping information acquisition method and the separated method to optimize the coded apertures in real applications, where 512 × 512 is the minimum spatial resolution. It is noted that the PPMCC matrix is an MP × MP dense matrix, though the MP × N structure matrix is highly sparse. Therefore, the memory requirement for the calculation and storage of the corresponding PPMCC matrix is ∼2 TB for a 512 × 512 image.

In this paper, a distributed approach based on block matrix multiplication is presented to estimate the sparse PPMCC matrix. Given a particular matrix *B*, $B^{T}B$ can be written as [18]

(11) $$B^{T}B=\left[\begin{array}{ccc} B_{1}^{T}B_{1} & \ldots & B_{1}^{T}B_{s}\\ \ldots & \ldots & \ldots \\ B_{s}^{T}B_{1} & \ldots & B_{s}^{T}B_{s} \end{array}\right],$$

where the matrices $B_{1},B_{2},\ldots ,B_{s}$ are the block matrices defined as $B={\left[B_{1}^{T},B_{2}^{T},\ldots ,B_{s}^{T}\right]}^{T}$.

Substituting Equation (10) into Equation (11) gives

(12) $$\begin{array}{cc} \Sigma & =\frac{1}{N-1}{\mathrm{WW}}^{T}\\ & =\frac{1}{N-1}\left[\begin{array}{ccc} W_{1}W_{1}^{T} & \ldots & W_{1}W_{s}^{T}\\ \ldots & \ldots & \ldots \\ W_{s}W_{1}^{T} & \ldots & W_{s}W_{s}^{T} \end{array}\right], \end{array}$$

where the matrices $W_{1},W_{2},\ldots ,W_{s}$ are the block matrices defined as $W={\left[W_{1}^{T},W_{2}^{T},\ldots ,W_{s}^{T}\right]}^{T}$. Since the nature of CS is to reconstruct the tomographic images from the fluctuation of the measurements, the variables in the structure matrix should be standardized. Therefore, Equation (12) is re-written as

(13) $$\Sigma =\frac{1}{N-1}\left[\begin{array}{ccc} H_{1}H_{1}^{T} & \ldots & H_{1}H_{s}^{T}\\ \ldots & \ldots & \ldots \\ H_{s}H_{1}^{T} & \ldots & H_{s}H_{s}^{T} \end{array}\right],$$

where $H_{i}=Z\left(W_{i}\right)$; i=1,2,…,s; and Z represents the standardization of the rows of the matrix. It is seen that the computation of the PPMCC matrix can be divided into s × s matrix multiplication of *s* smaller block matrices. Thus it can be assigned to s × s computers (or nodes in supercomputers), and each computer (node) only calculates the matrix multiplication of two smaller block matrices. If the number of computers (nodes) is less than $s^{2}$, the matrix multiplication of different block matrices can be performed using iterations of fewer computers (nodes). That is, $l_{0}$ iterations are required in each computer (node), the number of which is equal to $\left\lceil s^{2}/l_{0}\right\rceil$.

**Figure 3:**
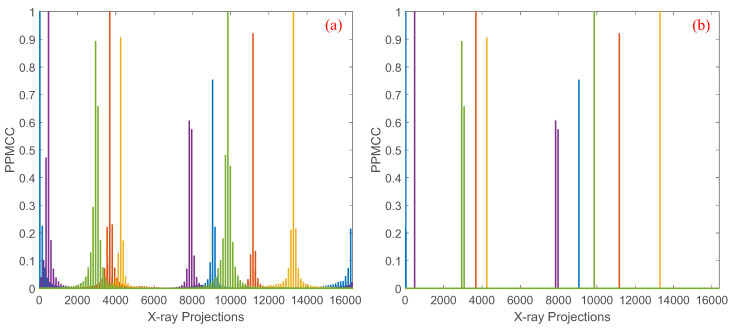
(**a**) shows the dense PPMCC matrix computed using all coefficients, and (**b**) shows the sparse PPMCC matrix obtained by applying a threshold of 0.5 for 128  ×  128 images.

As depicted in Equation (13), the PPMCC matrix can be calculated using distributed computing or multiple iterations with fewer computers based on block matrix multiplication. However, the matrix Σ exceeds the memory limitation and thus cannot be used in the algorithm solving Equation (9). In our previous work, it is demonstrated that the coefficient in the PPMCC matrix is determined by the intersections of the corresponding X-ray paths [14]. Therefore, the coefficients of two random paths, with high probability, are smaller than those of two neighboring X-ray paths and thus have fewer contributions to the representation of the structure property generated by the projection geometry. An example of the coefficients of five random X-ray paths with the other paths for $N=128^{2}$, M=128 and P=128 are depicted in Figure 3a. It is seen that the plot is highly structured, but most of the coefficients for a particular X-ray path are zero or almost zero. From the perspective of information theory, the coefficients can considered as the overlapping information, and non-zero entries represent the structure of the projections. Thus, the structure property of the PPMCC matrix can be sparsely represented by the highly structured coefficients. As depicted in Figure 3b, the structure property is estimated in the sparse representation with a threshold τ=0.5 compared with that in the dense representation in Figure 3a. Mathematically, the sparse representation of the block matrix multiplication $H_{i}H_{j}^{T}$, i=1,2,…,s, j=1,2,…,s, can be given by

(14) $$Q{\left(R\right)}_{r,t}=\left\{\begin{array}{cc} 0 & |R_{r,t}|<\tau \\ |R_{r,t}| & |R_{r,t}|\geq \tau \end{array}\right.$$

where $R_{r,t}=H_{i}H_{j}^{T}\left(r,t\right)$, r=1,2,…,MP/s, r=1,2,…,MP/s.

**Figure 4:**
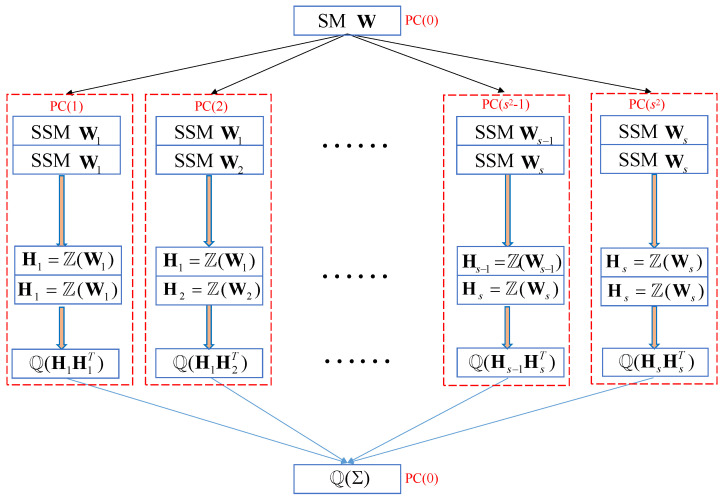
The flow diagram of sparse estimation of the PPMCC matrix.

The sparse estimation of the PPMCC matrix is summarized in Figure 4. First, the structure matrix is divided into *s* block structure matrices by rows. Then, the block structure matrices are paired, standardized and sequentially stored in $s^{2}$ computers (nodes). Next, the covariance matrix of the paired block sub-matrices is computed and sparsely estimated with a threshold τ. Finally, the sparse sub-matrices are combined sequentially to obtain the sparse PPMCC matrix.

### 3.2. Searching Algorithm

A variant of the locally optimal searching algorithm in [14] is presented to search the combinations of X-ray projections. It is a heuristic algorithm in which the selection of a particular X-ray projection is based on the distribution of the correlation coefficients of the selected projections. The key step in the algorithm is to determine the index of the minimum entry in the vector, q, which is the sum of the columns corresponding to the selected X-ray projections in the squared (dense or sparse) PPMCC matrix. However, the minimum entry often corresponds to several indexes, leading to a local optimum with high possibility in the heuristic algorithm. In [14], the errors of single-precision floating-point computations of the dense PPMCC matrix act as disturbance to avoid entering the local optimum, but the single-precision floating-point computations for very small coefficients in the sparse PPMCC matrix are estimated as 0. Thus, the heuristic searching, with high possibility, will enter the local optimum with a few iterations. A noise factor is used in the maximum index searching to reduce the possibility of entering the local optimum. The noise factor is given by $n=1\;\times \;10^{-3}\;random\left(\max\left(q\right)\right)$, where random represents the generation of a uniformly distributed random vector in the interval (0, 1). The searching algorithm is summarized in Algorithm 1.
**Algorithm 1** Local optimization of coded apertures**Require:** Sparse PPMCC matrix, Q(Σ);
**Require:** Number of iterations $t_{\max}=D$;
**Require:** $\mathrm{codes}\in R^{MP}$ and $l\in R^{MP}$
1:Compute the vector $q=\sum_{j=1}^{MP}Q{\left(\Sigma \right)}_{ij}^{2}$, where $q\in R^{MP}$2:$n=1e^{-3}random\left(\max\left(q\right)\right)$3:q←q+n4:Search the index $k_{1}$ with the minimal value in the vector q5:$\mathrm{codes}(k_{1})=1$, $l_{1}=k_{1}$6:**for** t=2 to $t_{\max}$ **do**7: Compute the vector $q=\sum_{j=l_{p}}Q{\left(\Sigma \right)}_{ij}^{2}$, where p=1,2,…,MP and $l_{p}\neq 0$8: $n=1e^{-3}random\left(\max\left(q\right)\right)$9: q←q+n10: Search the index $k_{t}$ with the minimal value in the vector q11: $\mathrm{codes}(k_{t})=1$, $l\left(t\right)=k_{t}$12:**end for**13:**return** codes


## 4. Simulations

To further study the proposed coded aperture optimization approach, numerical simulations with a fan-beam projection geometry were performed. The coded apertures with 256 × 1 pixels were placed in front of the X-ray source, and the coded projections were measured by a 2D 256 × 1 detector. Note that the elements on the coded apertures have one-to-one correspondence to those on the detector. The geometric lengths of the flat detector, the source-to-object distances and the source-to-detector distances are 40 cm, 40 cm and 80 cm, respectively. The ASTRA Tomography Toolbox [19] was used to obtain the discrete-to-discrete structure matrix W, and the open access dataset “Walnut Phantom” with 128 × 128 pixels was used as the original object [20]. That is, N=128, and P=M=256. The gradient projection for sparse reconstruction (GPSR) algorithm was used to reconstruct the tomographic images where the 2D Haar wavelet basis was used as the sparse basis [21]. Note that the reconstruction is the average of 10 iterations in the random cases. The coded aperture optimization and reconstruction were performed in a desktop architecture with an Intel Core i7 3.6 GHz processor, with 64 GB RAM, using MATLAB 2017a. The peak signal-to-noise ratio (PSNR) and structural similarity (SSIM) are used to evaluate the reconstructed images. The PSNR is given by

(15) $$\mathrm{PSNR}=20\cdot \log_{10}\left(\frac{f_{\max}}{\sqrt{\mathrm{MSE}}}\right),$$

where $\mathrm{MSE}=\frac{{\sum \sum \left(f-\hat{f}\right)}^{2}}{N^{2}}$, and $\hat{f}$ and $f_{\max}$ denote the reconstructed image and its maximum value, respectively. The SSIM is given by

(16) $$\mathrm{SSIM}\left(x,y\right)=\frac{\left(2\mu_{x}\mu_{y}+c_{1}\right)\left(2\sigma_{xy}+c_{2}\right)}{\left(\mu_{x}^{2}+\mu_{y}^{2}+c_{1}\right)\left(\sigma_{x}^{2}+\sigma_{y}^{2}+c_{2}\right)},$$

where $\mu_{x}$ and $\mu_{y}$ are the mean of *x* and *y*, respectively. $\sigma_{x}^{2}$ and $\sigma_{y}^{2}$ are the variance of *x* and *y*, respectively. $\sigma_{xy}$ is the covariance of *x* and *y*, and $c_{1}$ and $c_{2}$ are constants.

### 4.1. 128 × 128 Images

**Figure 5:**
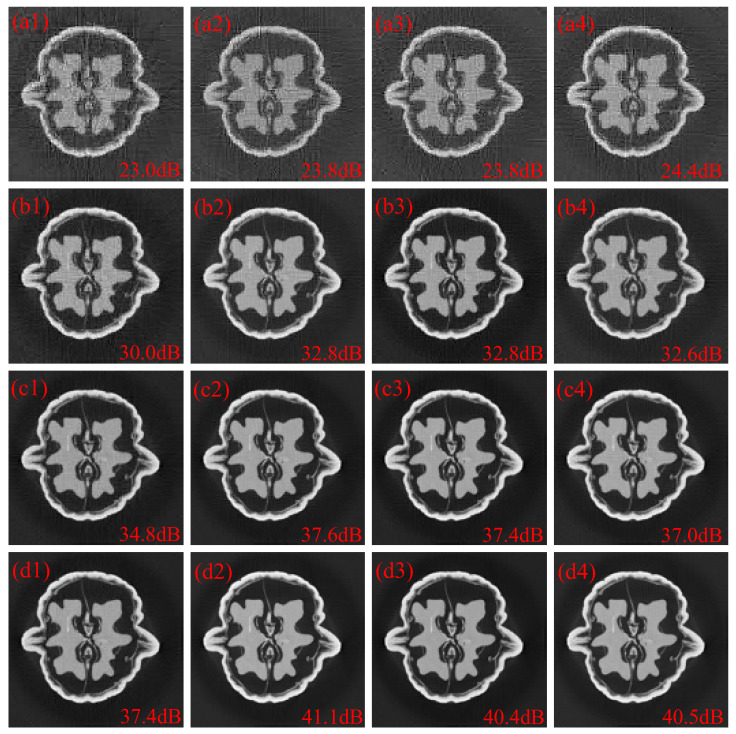
(**a1**–**d1**) are the 128 × 128 reconstructed images with random coded apertures at 30%, 50%, 70% and 90% sub-sampling rates, respectively. (**a2**–**d2**) are the 128 × 128 reconstructed images with optimized coded apertures based on SPCA at 30%, 50%, 70% and 90% sub-sampling rates, respectively. (**a3**–**d3**) are the 128 × 128 reconstructed images with optimized coded apertures based on the SCME method with τ=0.1 at 30%, 50%, 70% and 90% sub-sampling rates, respectively. (**a4**–**d4**) are the 128 × 128 reconstructed images with optimized coded apertures based on the SCME method with τ=0.2 at 30%, 50%, 70% and 90% sub-sampling rates, respectively.

The images with 128 × 128 pixels were reconstructed from projections acquired at 256 view angles using a detector with 256 pixels. That is, N=128, and P=M=256. The reconstructed 128 × 128 images with random coded apertures, optimized coded apertures based on the SPCA method and optimized coded apertures based on the sparse correlation matrix estimation (SCME) method are depicted in Figure 5. The letters “a”, “b”, “c” and “d” represent 30%, 50%, 70% and 90% sub-sampling rates, respectively. The labels “1”, “2”, “3” and “4” refer to random coded apertures, optimized coded apertures based on the SPCA method, optimized coded apertures based on the SCME method with τ=0.1 and optimized coded apertures based on the SCME method with τ=0.2, respectively. Reconstructions obtained with optimized apertures show fewer artifacts than those produced with random apertures. At sub-sampling rates of 30%, 50%, 70% and 90%, the corresponding PSNR gains over the random baseline are approximately 1.0 dB, 2.7 dB, 2.5 dB and 3.3 dB. As depicted in Figure 5(a1–a4,b1–b4), the sparsity estimation has little effect on the optimization performance. And it seems to help the searching algorithm to jump from local optimal solution at low sub-sampling rates as shown in Figure 5(a4). On the other hand, the sparsity estimation does not perform as well as the SPCA method at a high sub-sampling rate (90%) where all correlation coefficients are used.

The PSNR and SSIM for 128 × 128 images at different sub-sampling rates using random, SPCA and SCME strategies are shown in Table 1. As depicted in Table 1, the structure of the “Walnut” images could be well reconstructed (PSNR > 30 dB and SSIM > 0.94) at sub-sampling rates greater than 50%, where the coded aperture transmittance is 12.5%, leading to 87.5% radiation dose reduction. Compared with random coded apertures, the optimized coded apertures significantly improve the reconstruction accuracy of the attenuation coefficient at a particular pixel and thus lead to fewer artifacts. Both the SPCA method and SCME method have fewer artifacts and quantitatively more than 2.5 dB PSNR gains over random coded apertures. It is also seen that the SCME method performs as well as the SPCA method even with coefficients smaller than 0.27% in the PPMCC matrix in most cases.

### 4.2. 256 × 256 Images

**Figure 6:**
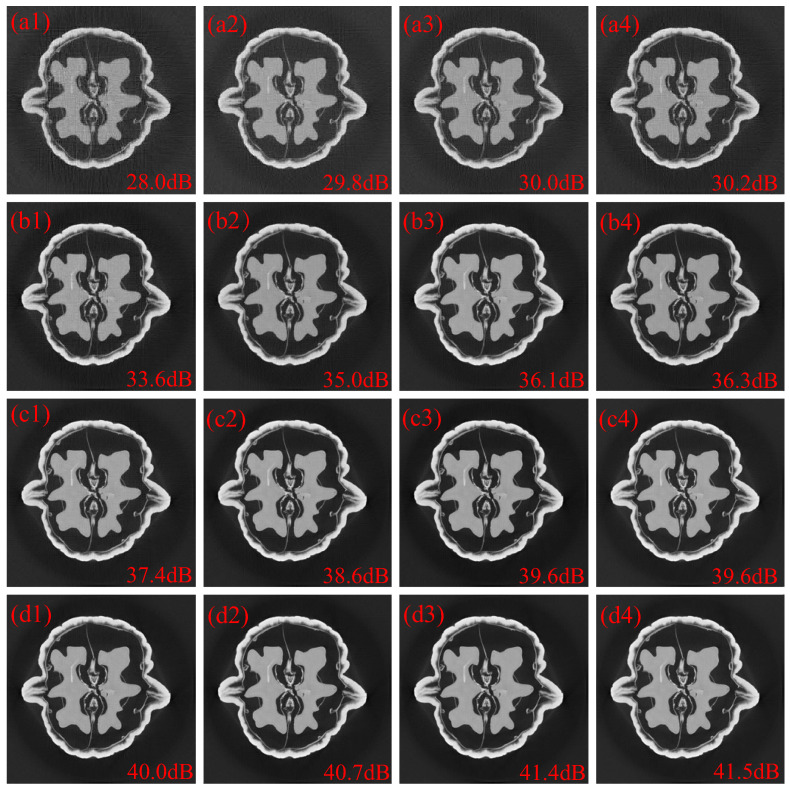
(**a1**–**d1**) are the 256 × 256 reconstructed images with random coded apertures at 30%, 50%, 70% and 90% sub-sampling rates, respectively. (**a2**–**d2**) are the 256 × 256 reconstructed images with optimized coded apertures based on SPCA at 30%, 50%, 70% and 90% sub-sampling rates, respectively. (**a3**–**d3**) are the 256 × 256 reconstructed images with optimized coded apertures based on the SCME method with τ=0.1 at 30%, 50%, 70% and 90% sub-sampling rates, respectively. (**a4**–**d4**) are the 256 × 256 reconstructed images with optimized coded apertures based on the SCME method with τ=0.2 at 30%, 50%, 70% and 90% sub-sampling rates, respectively.

Additional simulations using the images with 256 × 256 pixels were performed, where the projections from 512 view angles were measured by a detector with 512 pixels. That is, N=256, and P=M=512. Note that, in this scenario, the memory requirement for the correlation matrix in the SPCA method exceeds the regular platform, and thus a separated method where the structure matrix is divided into four sub-matrices was used to alleviate the tradeoff between the memory requirement and large structure matrix. The reconstructed 256 × 256 images with random coded apertures, optimized coded apertures based on the separated SPCA method and optimized coded apertures based on the SCME method are depicted in Figure 6. The letters “a”, “b”, “c” and “d” represent 30%, 50%, 70% and 90% sub-sampling rates, respectively. The numbers “1”, “2”, “3” and “4” represent random coded apertures, optimized coded apertures based on the separated SPCA method, optimized coded apertures based on the SCME method with τ=0.1 and optimized coded apertures based on the SCME method with τ=0.2, respectively. It is seen that the images reconstructed with the SCME method have fewer artifacts than those with random coded apertures and optimized coded apertures with the separated SPCA method. Compared with reconstructions using random coded apertures, the PSNR gains of reconstruction using optimized coded apertures based on the separated SPCA method and SCME method are ∼0.8 dB, ∼1.4 dB, ∼1.2 dB and ∼0.7 dB, and ∼2.1 dB, ∼2.6 dB, ∼2.2 dB and ∼1.5 dB, at 30%, 50%, 70% and 90% sub-sampling rates, respectively. Compared with the separated SPCA method, the PSNR gain of the SCME method is greater than 1dB. As depicted in Figure 5 and Figure 6, the sparsity estimation in the cases of 256 × 256 images has similar performanceto the SPCA method for 128 × 128 images without separated operation.

The PSNR and SSIM for 256 × 256 images at different sub-sampling rates using the random and separated methods and SCME strategies are shown in Table 2. As depicted in Table 2, the structure of the “Walnut” images could be well reconstructed (PSNR > 30 dB and SSIM > 0.94) at greater than 40% sub-sampling rates, where the coded aperture transmittance is 10%, leading to 90% radiation dose reduction. It is seen that the optimization performance of the SPCA method degrades using the separated method. Quantitatively, the PSNR gain decreases from 2.8 dB to 1.2 dB. On the other hand, the PSNR gain of the proposed SCME method is 2.2dB, and coefficients smaller than 0.02% are used in the PPMCC matrix.

### 4.3. 512 × 512 Images

**Figure 7:**
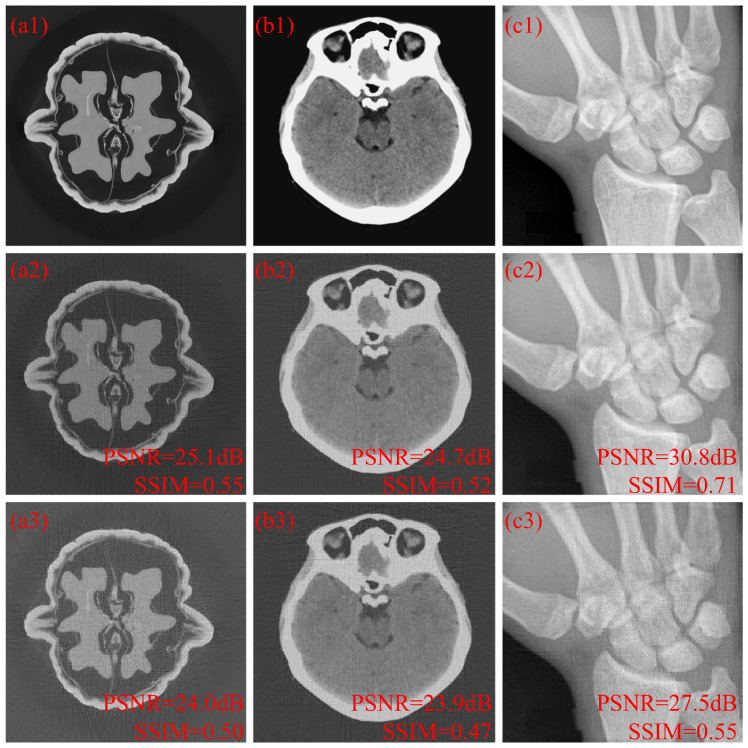
(**a1**–**a3**) are the original image, reconstructed image with random coded apertures and reconstructed image with optimized coded apertures based on the SCME method of “Walnut”. (**b1**–**b3**) are the original image, reconstructed image with random coded apertures and reconstructed image with optimized coded apertures based on the SCME method of “Brain”. (**c1**–**c3**) are the original image, reconstructed image with random coded apertures and reconstructed image with optimized coded apertures based on the SCME method of “Bone”.

The simulation scenario for the images with 512 × 512 pixels was performed with 256 projection view angles. The coded projections were measured by a detector with 1024 pixels. That is, N=512, P=256 and M=1024. The SPCA method and the separated SPCA method cannot be used to optimize the coded apertures due to the expensive computational cost of execution time and memory requirement. Three different tomographic images, “Walnut Phantom”, “Brain” and “Bone”, were used in the simulations. The reconstructed 512 × 512 images at 10% sub-sampling rates with random coded apertures and optimized coded apertures based on the SCME method are depicted in Figure 7. The letters “a”, “b” and “c” represent “Walnut Phantom”, “Brain” and “Bone”, respectively. The numbers “1”, “2” and “3” represent original images, random cases and optimized cases based on the SCME method with τ=0.2, respectively. The coefficients used in the sparse PPMCC matrix are smaller than 0.02%, and the images reconstructed with the SCME method have far fewer artifacts than those with random coded apertures. As shown in the reconstructed images with many details, ”Walnut Phantom” and “Brain”, the recovery leads to fewer artifacts and more smooth images. On the other hand, the recovery of “Bone”, with more structures, leads to higher contrast and more clear structure. Quantitatively, the average PSNR gain and SSIM gain are 1.7 dB and 0.1, respectively.

## 5. Conclusions

In this paper, a coded aperture design is introduced based on sparse covariance matrix estimation. It is a variant of the series of approaches that minimize the information loss or maximize the information acquisition. The proposed method significantly reduces the computational load of the PPMCC matrix to $1/s^{2}$, and coefficients smaller than 0.02% are used in the optimization. The PSNR and SSIM of the reconstructed imagery are used in the performance analysis of the proposed coded aperture optimization algorithms. Numerical experiments with simulated datasets show PSNR gains up to 3.3 dB and SSIM gains up to 0.16 compared with those of random cases for 512 × 512 images. The proposed method is broadly applicable and can be adapted to alternative CAXCT geometries, as well as to other coded aperture imaging systems, including coded aperture spectral imaging architectures [22]. 

## Figures and Tables

**Table 1:** PSNR and SSIM for 128 × 128 images at different sub-sampling rates using random, SPCA and SCME strategies.

	SR	30%	40%	50%	60%	70%	80%	90%	100%
Random Method	PSNR	22.99	27.49	29.91	32.41	34.77	35.90	37.37	38.99
	SSIM	0.78	0.91	0.94	0.97	0.98	0.98	0.99	0.99
SPCA Method	PSNR	23.75	**29.73**	**32.83**	**35.53**	37.59	39.69	**41.14**	**41.82**
	SSIM	0.79	**0.94**	**0.97**	**0.98**	0.99	0.99	**0.99**	**0.99**
SCME τ=0.1	PSNR	23.82	29.70	32.76	35.33	37.38	38.97	40.42	41.54
	SSIM	0.79	0.94	0.97	0.98	0.99	0.99	0.99	0.99
SCME τ=0.2	PSNR	**24.43**	29.53	32.61	35.08	37.04	39.01	40.49	41.56
	SSIM	**0.83**	0.93	0.97	0.98	0.99	0.99	0.99	0.99
SCME τ=0.3	PSNR	22.84	29.69	32.59	35.51	**37.61**	39.15	40.37	41.51
	SSIM	0.75	0.94	0.97	0.98	**0.99**	0.99	0.99	0.99
SCME τ=0.4	PSNR	23.25	29.33	32.41	35.18	37.10	**39.69**	40.68	41.76
	SSIM	0.77	0.94	0.97	0.98	0.99	**0.99**	0.99	0.99
SCME τ=0.5	PSNR	22.15	28.00	32.11	34.87	37.21	38.52	40.39	41.56
	SSIM	0.73	0.91	0.96	0.98	0.99	0.99	0.99	0.99

**Table 2:** PSNR and SSIM for 256 × 256 images at different sub-sampling rates using random, SPCA and SCME strategies.

	SR	30%	40%	50%	60%	70%	80%	90%	100%
Random Method	PSNR	28.07	31.11	33.58	35.54	37.41	38.63	39.93	41.20
	SSIM	0.89	0.94	0.97	0.98	0.98	0.99	0.99	0.99
Separated Method	PSNR	29.84	32.60	35.03	37.04	38.63	39.78	40.68	41.46
	SSIM	0.93	0.96	0.97	0.98	0.99	0.99	0.99	0.99
SCME τ=0.2	PSNR	30.07	**33.65**	36.12	38.05	39.61	40.52	41.37	42.07
	SSIM	0.92	**0.97**	0.98	0.99	0.99	0.99	0.99	0.99
SCME τ=0.3	PSNR	**30.23**	33.61	**36.32**	**38.25**	39.56	40.54	41.45	42.21
	SSIM	**0.93**	0.97	**0.98**	**0.99**	0.99	0.99	0.99	0.99
SCME τ=0.4	PSNR	29.82	32.87	35.49	37.66	**39.64**	**41.00**	**41.68**	**42.41**
	SSIM	0.93	0.96	0.98	0.98	**0.99**	**0.99**	**0.99**	**0.99**
SCME τ=0.5	PSNR	29.27	32.52	35.26	37.39	39.22	40.41	41.32	42.30
	SSIM	0.92	0.96	0.98	0.98	0.99	0.99	0.99	0.99

## Data Availability

Dataset available on request from the authors.
